# Significant changes in gut microbiota and SCFAs among patients with newly diagnosed acute myeloid leukemia

**DOI:** 10.3389/fmicb.2025.1559033

**Published:** 2025-04-01

**Authors:** Shujuan An, Xia Gong, Long Zhao, Jinli Jian, Yuancheng Guo, Xiaoxiao Yang, Hongjia Sun, Yang Li, Bei Liu

**Affiliations:** ^1^The First Clinical Medical College, Lanzhou University, Lanzhou, China; ^2^Department of Medical Laboratory, The First Hospital of Lanzhou University, Lanzhou, China; ^3^Department of Haematology, The First Hospital of Lanzhou University, Lanzhou, China

**Keywords:** acute myeloid leukemia, gut microbiota, butyric acid, *Enterococcus*, *Enterococcus faecium*

## Abstract

The purpose of this study was to identify whether the gut microbiota and metabolites of newly diagnosed acute myeloid leukemia (AML) patients displayed specific characteristic alterations and whether these changes could be used as potential biomarkers for predicting the disease. Notably, the gut microbiota and metabolites of AML patients exhibited significant structural and quantitative alterations at the time of their initial diagnosis. Beneficial bacteria, including *Faecalibacterium*, *Collinsella*, *Lacticaseibacillus*, and *Roseburia*, as well as butyric acid and acetic acid, were found to be considerably reduced in newly diagnosed AML patients. In contrast, *Enterococcus* and *Lactobacillus*, especially *Enterococcus*, were significantly enriched. Further investigation indicated that *Enterococcus* could serve as a potential intestinal marker, showing a strong negative correlation with the levels of acetic and butyric acid. Importantly, assays aimed at identifying AML demonstrated that *Enterococcus*, butyric acid, and acetatic acid exhibited excellent predictive effectiveness. Colonizing *Enterococcus* from patients were isolated for pathogen investigation, which revealed that these bacteria possess several strong virulence factors and multiple drug-resistance gene characteristics. Therefore, we speculate that the increase of *Enterococcus* may contribute to the development and progression of AML.

## Introduction

Recent advancements in multi-omics technology have generated significant interest in exploring the role of gut microbiota in the treatment and prognosis of acute myeloid leukemia (AML). Research indicates that low baseline microbiota diversity is a strong independent predictor of infection during AML induction chemotherapy (IC). Specifically, prolonged use of carbapenems (over 72 h) is associated with significantly lower *α*-diversity, while higher baseline levels of *Porphyromonadaceae* seem to offer protection against infection. Therefore, assessing gut microbiota can aid in stratifying infection risk, and optimizing antibiotic dosing may help reduce subsequent infectious complications in AML patients (Galloway-Peña et al., 2016; Galloway-Peña et al., 2020). Moreover, gut microbiota analysis can identify patients at high-risk of developing bloodstream infections (BSIs), with the gut microbiota of *Barnesiellaceae*, *Christensenellaceae*, and *Faecalibacterium* being significantly reduced in high-risk patients, while *Erysipelotrichaceae* and *Veillonella* were significantly increased, which may be a promising avenue for future research (Montassier et al., 2016). Disruptions in microbiota, characterized by reduced diversity and domination by specific microbiota such as *Enterococcus, Klebsiella, Escherichia, Staphylococcus,* and *Streptococcus* during hematopoietic stem cell transplantation (HSCT), have been linked to higher mortality rates (Peled et al., 2020). Additionally, it has been observed that both *Faecalibacterium* and *Roseburia* were significantly reduced in the gut microbiota of AML patients. These reductions are negatively correlated with peripheral leukocyte levels and the percentage of bone marrow (BM) blast cells and may contribute to decreased butyric acid production, which impairs the intestinal barrier and promotes the development of AML (Wang et al., 2022). Another study confirmed that the microbiota of AML patients at baseline was enriched in frequently pathogenic species (e.g., *Enterococcus*, *Staphylococcus*) while being depleted in *Faecalibacterium* and *Ruminococcus*, both known butyric acid producer (Rashidi et al., 2023). Intensive treatment of AML can transiently impairs gut barrier function and induce persistent changes in the composition and metabolic activity of the gut microbiota, alterations that are associated with cachectic symptoms (Pötgens et al., 2023). However, it is important to note that the majority of studies have focused on the longitudinal comparisons encompassing pre-and post-chemotherapy assessments, pre-and post-transplantation evaluations, BSI, and prognostic predictions. Given that AML patients commonly receive multiple antibiotics during IC, significant disruptions to their gut microbiota are inevitable (Rashidi et al., 2022; Ramirez et al., 2020).

Is it possible that the gut microbiota was already structurally altered in patients with newly diagnosed AML? Metagenomic analysis has demonstrated its potential to distinguish healthy individuals from cancer patients and serve as diagnostic bacterial markers in various diseases. For instance, *Bacteroides fragilis* and *Fusobacterium nucleatum* have been identified in colorectal cancer (Cheng et al., 2020), while *Akkermansia muciniphila*, *Rikenellaceae* and *Bacteroides* are linked to non-small cell lung cancer (Vernocchi et al., 2020). Therefore, this study aims to hypothesize whether detection of characteristically altered gut microbiota and associated metabolites could aid in screening for AML.

In this study, we performed a comparative analysis of the gut microbiota and metabolites between healthy individuals and newly diagnosed AML patients, organized along a horizontal axis. Additionally, we also investigated the potential of gut microbiota and metabolites from newly diagnosed AML patients as intestinal biomarkers, while also exploring their molecular epidemiology of *Enterococcus*.

## Materials and methods

### Participants and clinical characteristics

We enrolled 32 consecutive patients newly diagnosed with AML in the study. All patients were undergoing their first cycle of IC from March to September 2023 at the Department of Hematology at the First Hospital of Lanzhou University. The diagnosis of AML was based on the World Health Organization (WHO) classification criteria for myeloid neoplasms, which defines it as a greater than 20% presence of myeloid blasts in circulation and/or bone marrow examination (Newell and Cook, 2021; Rattanathammethee et al., 2020). Participants who met any of the following criteria were excluded: (a) recent antibiotic treatment within the past month, (b) recent use of probiotics within the past month, (c) comorbidities involving other cancers, and (d) a diagnosis of acute promyelocytic leukemia. Additionally, we recruited healthy control subjects from outpatient clinics and medical examination centers during the same period to exclude individuals with metabolic, digestive, cardiovascular, endocrine, and neurological disorders. Initial demographic and hematological parameters of all participants were obtained by reviewing the electronic medical records.

### Sample collection

Fecal samples were obtained from all participants, and placed into sterile preservation tubes, which were then promptly stored at −80°C for further analysis of gut microbiota and metabolite. All biological sampling and data collection was completed prior to the administration of any treatment.

### Fecal 16S rRNA sequencing

#### Bacterial stool DNA extraction

Total Bacterial genomic DNA was extracted using the CTAB method, and the quality of DNA extraction was assessed using SDS and UV spectrophotometry. The V3-V4 hypervariable region of the 16S ribosomal RNA gene was amplified using a primer set corresponding to primers 341\u00B0F (5’-CCTACGGGNGGCWGCAG-3′) and 805 R (5’-GACTACHVGGGTATCTAATCC-3′). The PCR products were purified with AMPure XT beads (Beckman Coulter Genomics, Danvers, MA, USA), and quantified using a Qubit (Invitrogen, USA). The purified PCR products were then recovered with AMPure XT beads. Amplicon pools were prepared for sequencing, and both the size and quantity of the amplicon library were evaluated using Walker et al., 2023 Bioanalyzer (Agilent, USA) as well as with the Library Quantification Kit for Illumina (Kapa Biosciences, Woburn, MA, USA). Finally, the libraries were sequenced on NovaSeq platform PE250.

#### Analysis of sequencing data

For the bipartite data obtained by sequencing, it is first necessary to split the data of the samples based on the barcode information and remove the splice and barcode sequences. By data splicing and filtering: (a) Remove primer sequences and balanced base sequences from RawData. (Software: cutadapt, v1.9); (b) Splicing and merging each pair of paired-end reads into one longer tag according to the overlap region (Software: FLASH, v1.2.8); (c) Performing quality scanning on sequencing reads, with the scanning window defaulted to 100 bp, and when the average quality value is below 20, the read is truncated from the start of the window to the 3′ termination. (Software: fqtrim); (d) Remove sequences whose length after truncation is less than 100 bp; (e) Remove sequences whose N content after truncation is more than 5%; (f) Remove chimeric sequences. (Software: Vsearch, v2.3.4). Then quite divisive amplicon denoising algorithm 2 (DADA2) denoise-paired for length filtering and denoising. Amplicon Sequence Variants (ASVs) feature sequences and ASVs feature abundance tables were obtained and singletons ASVs were removed.

The *α*-diversity and *β*-diversity analysis were performed based on the obtained ASVs feature sequences and ASVs feature abundance tables. Where α-diversity analysis was mainly assessed by chao1 and Shannon index (calculated based on ASVs levels). Differences in α-diversity metrics were tested using the non-parametric Mann–Whitney test. β-diversity analyses usually start by calculating the distance matrix between samples based on Bray-Curtis metrics, which is mainly done by Principal coordinates analysis (PCoA) and Nonmetric Multidimensional Scaling (NMDS) methods to observe the differences between the samples.

### Targeted metabolomics profiling and analysis

To characterize the metabolomic profile of short-chain fatty acids (SCFAs) in fecal samples from AML patients, we conducted targeted metabolomics using a SHIMADZU GC2030-QP2020 NX gas chromatography-mass spectrometer, equipped with an HP-FFAP capillary column. The samples were first thawed on ice, pretreated with 50% H_2_SO4 and extraction solution (25 mg/L of internal standard 2-methylvaleric acid and methyl tert-butyl ether) to extract metabolites, and then the appropriate amount of the analytes to be measured was transferred to the HP-FFAP capillary for quantitative detection. The standard curve was plotted according to the detection level of the standards, and then the concentration of the analyte was calculated according to the standard curve.

### Isolation, identification, and antimicrobial susceptibility testing of intestinal bacteria colonizing newly diagnosed AML patients

Clinical fecal specimens were initially cultured on Columbia Blood Agar plates to promote bacterial growth. Species identification was carried out using matrix-assisted laser desorption ionization time-of-flight mass spectrometry (MALDI-TOF MS, bioMerieux, France). This method relies on detecting the mass-to-charge ratios of bacterial ribosomal proteins and is widely used in microbiology applications (Ashfaq et al., 2022). Additionally, the minimum inhibitory concentration (MIC) of various antimicrobial agents was determined using the VITEK 2 Compact system (bioMérieux, France) (Sengupta et al., 2021). Quality control for antimicrobial susceptibility testing (AST) was performed with *Staphylococcus aureus* ATCC 25923 and *Enterococcus faecalis* (*E. faecalis*) ATCC 29212. The MIC results were evaluated based on the interpretation criteria established by the Clinical and Laboratory Standards Institute CLSI M100 (Pierce et al., 2023).

### Sequencing of gut colonizing *Enterococcus. faecium* of newly diagnosed AML patients

The molecular characterization of *Enterococcus faecium* (*E. faecium*) isolates ware conducted using Next Generation Sequencing (NGS) (Wensel et al., 2022; Larson et al., 2023). For genomic DNA extraction, a plant Genomic DNA kit (Tiangen, DP305) was utilized. The DNA concentration was measured with a NanoDrop™ 2000 (Thermo Scientific, Waltham, MA) spectrophotometer and verified through agarose gel electrophoresis. The libraries were prepared using the TruePrep™ DNA Library Prep Kit V2 for Illumina (Vazyme) and were subsequently sequenced on an Illumina NovaSeq platform (Illumina Inc., San Diego, CA, USA).

The raw sequenced reads are first quality-checked using FastQC (Version 0.11.9) and MultiQC (Version 1.10.1). Once the raw reads pass quality control, adapter sequences in the samples are trimmed using Trim Galore (Version 0.6.6). The trimmed reads are then assembled using the *de novo* assembly tool called Unicycler (Version 0.4.5).^1^ The assembled genomic data can be analyzed using a variety of bioinformatics tools. Antibiotic resistance (AMR) genes from the Comprehensive Antibiotic Resistance Database (CARD)^2^ were used for identification. We searched the Public Database for Molecular Typing and Microbial Genome Diversity (PubMLST)^3^ for sequence types (ST) of all isolates. We also analyzed the virulence factors (VFs) of *E. faecium* according to the Virulence Factor Database (VFDB).^4^

### Statistical analysis

GraphPad Prism 10.2.0 (La Jolla, California, USA) was applied for graph development. Statistical analysis was performed using the SPSS software package V.26 (SPSS, Chicago, USA). The Shapiro–Wilk test was utilized for normality tests. Normally distributed data are presented as mean ± standard deviation (M ± SD) and analyzed by Student’s t-test or unpaired t-tests. Otherwise, non-normally distributed continuous variables are expressed as median (interquartile range, IQR) and performed using the Mann–Whitney U test. Venn diagrams were utilized to display areas of overlap in the gut microbiota between the AML and HC groups. A random forest plot was used to demonstrate the importance of different genera of bacteria. Univariate analysis based on the relative abundances of microbiota was performed using the linear discriminant analysis effect size (LEfSe) method. Receiver operating characteristic curve (ROC) analysis is a powerful tool for assessing the performance of gut microbiota and metabolites in predicting AML. Spearman correlations were constructed to analyze correlations between gut microbiota and metabolites and clinical characteristics. All significance tests were two-sided, and *p < 0.05, **p < 0.01, and ***p < 0.001 were considered statistically significant.

## Results

### Participant characteristics

We recruited 32 patients with newly diagnosed AML, comprising 16 males and 16 females, with an average age of 51 years. Additionally, we included 30 healthy control (HC) subjects, consisting of 18 males and 12 females, with an average age of 56 years during a parallel period. We ensured that there were no significant differences in age (*p* = 0.2232), sex (*p* = 0.4374), height (*p* = 0.483), and weight (*p* = 0.674) between the two cohorts, and excluded them from interfering with the gut microbiota to ensure comparability. The clinical characteristics of the 32 newly diagnosed AML patients are outlined in Table 1, while the initial demographic and hematological parameters that exhibited statistically significant differences between all patients and the 30 healthy controls are delineated in Table 2. According to 2022 European LeukemiaNet guidelines (DiNardo et al., 2023), the 32 patients were classified into two risk categories: 10 with favorable risk and 20 with unfavorable risk. The top 5 genes with the highest frequency of mutations were as follows: FLT3-ITD (34.38%), NRAS (25%), NPM1 (15.83%), WT1 (15.83%), and ASXL1 (15.83%).

**Table 1:** Clinical characteristics of newly diagnosed AML patients.

Clinical characteristics	Number
Age at baseline, (M ± SD)y	50.63 ± 15.49
Sex	
Male (%)	16 (50%)
Female (%)	16 (50%)
Baseline WBC count, median (range), (х10^9^/L)	20.11 (10.12–43.18)
Percentage of myeloid blasts, median (range), (%)	59.7 (27–86)
Genetic mutation (*N*, %)	
FLT3-ITD	11 (34.38)
NRAS	8 (25%)
NPM1	5 (15.63)
WT1	5 (15.63)
ASXL1	5 (15.63)
CEBPA bZIP	4 (12.5)
IDH2	4 (12.5)
PTPN11	3 (9.38)
FLT3-TKD	3 (9.38)
TP53	2 (6.25)
KRAS	2 (6.25)
KIT	2 (6.25)
Others^*^	13 (40.63)
Risk category (*N*, %)	
Favorable	10 (31.25)
Un-favorable (Intermediate and Adverse)	20 (62.5)
Missing data	2 (6.25)

* WBC, white blood cells; Others: DNMT3A; CSF3R; IDH1; MLLT3: KMT2A; ASXL3; CSMD1; JAK3; JAK2; SRSF; TET2; AML1-ETO (RUNX1-RUNX1T1); ABL1; U2AF1; ETV.

**Table 2:** Participant characteristics.

Participant characteristics	HC (*n* = 30)	AML (*n* = 32)	*p*-value
Sex (male; female)	18(male);12(female)	16(male);16(female)	0.4374
Age, years (mean ± SD)	55.6 ± 10.1^a^	50.6 ± 15.5^a^	0.2232
Body temperature (°C)	36.6 (36.5–36.63)^b^	36.6 (36.5–36.85)^b^	0.158
Height (cm)	167 ± 6.5^a^	166 ± 6.9^a^	0.483
Weight (kg)	63.2 ± 8.9^a^	62.1 ± 11.1^a^	0.674
WBC (х10^9^/L)	5.82 ± 2.00^a^	20.11 (10.12–43.18)^b^	<0.001
NEUT (%)	60.37 ± 9.94^a^	22.2 (16.95–45.55)^b^	<0.001
MONO (%)	5.9 (5.4–6.4)^b^	19.85 (4.25-49)^b^	0.005
RBC (х10^12^/L)	4.75 ± 0.34^a^	2.35 ± 0.73^a^	<0.001
HB (g/L)	148. ± 12.27^a^	72.76 ± 17.74^a^	<0.001
PLT (х10^9^/L)	200 ± 60^a^	42 (29-78)^b^	<0.001
PT (sec)	11.25 (10.86–11.5)^b^	13.594 ± 1.86^a^	<0.001
APTT (sec)	31.35 (29.88–33.3)^b^	29.32 ± 34.16^a^	0.0077
FIB (g/L)	2.76 ± 0.62^a^	3.57 (2.92–4.32)^b^	<0.001
DD (ug/ml)	0.26 (0.16–0.51)^b^	1.39 (0.88–3.83)^b^	<0.001
FDP (ug/ml)	0.71 (0.58–1.34)^b^	3.89 (2.04–8.95)^b^	<0.001
DBIL (μmol/L)	2.89 ± 0.85^a^	3.94 ± 2.20^a^	0.0294
ALB (g/L)	45.39 ± 2.79^a^	38.09 ± 4.51^a^	<0.001
TG (mmol/L)	3.81 ± 0.76^a^	2.96 (2.31–3.30)^b^	<0.001
LDL (mmol/L)	2.52 ± 0.51^a^	1.9 (1.4–2.18)^b^	<0.001
HDL (mmol/L)	1.19 ± 0.26^a^	0.69 ± 0.23^a^	<0.001
CK (U/L)	94.5 (75–148.75)^b^	37 ± 18.91^a^	<0.001
LDH (U/L)	186.44 ± 42.44^a^	463 (390.5–720.5)^b^	<0.001
PCT (ng/ml)	0.02 ± 0.01^a^	0.17 (0.09–0.29)^b^	<0.001
CRP (mg/L)	0.55 (0.26–2.45)^b^	32.34 (15.3–61.84)^b^	<0.001

^a^normal distribution and variance-aligned, M ± SD, unpaired *t*-test. ^b^abnormal distribution, median (interquartile spacing IQR), Mann–Whitney test. NEUT, neutrophil percentage; MONO, monocytes percentage; RBC, red blood cells; HB, hemoglobin; PLT, platelet; PT, prothrombin time; APTT, activated partial thromboplastin time; FIB, fibrinogen; DD, DD dimer; FDP, fibrinogen degradation products; DBIL, direct bilirubin; ALB, albumin; TG, triglyceride; LDL, low-density lipoprotein; HDL, high-density lipoprotein; CK, creatine kinase; LDH, lactate dehydrogenase; PCT, procalcitonin; CRP, c-reactive protein.

### Characterizations of gut microbiota diversity in newly diagnosed AML patients

**Figure 1:**
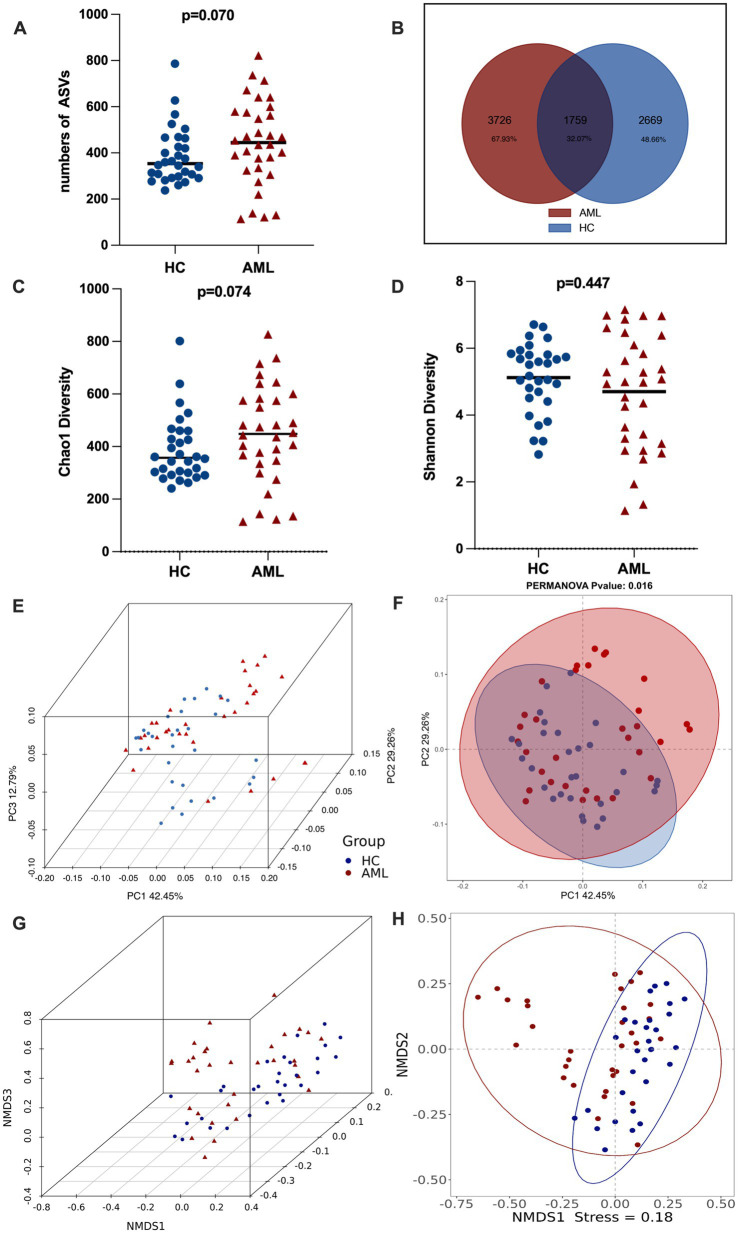
The ASV sequences and gut microbiota diversity in newly diagnosed AML patients and HC group. **(A)** Standard Boxplots of ASVs in AML and HC groups (non-normal distribution, Mann Whitney test). **(B)** Venn diagram of the observed AVSs in AML and HC (shared microorganisms: 1759, 32.07%; AML specific microorganisms: 3726, 67.93%). **(C,D)** A nonparametric test was used to compare the Chao 1 (non-normal distribution, Mann Whitney test) and Shannon index (a normal distribution with uneven variance, Mann–Whitney test). **(E,F)** PCoA 3D and 2D of faecal microbiota in AML and HC. The significance of two separated clusters was measured with the Adonis test (*p* = 0.016). **(G,H)** NMDS 3D and 2D of fecal microbiota in AML and HC. The coefficient of coercion (stress) is used to measure the merit of the NMDS analysis results, and it is usually considered that when stress<0.2, it can be represented by a two-dimensional dot plot of NMDS, which is graphically interpretable.

To investigate the changes in gut microbiota among newly diagnosed AML patients, we utilized ASVs (Rashidi et al., 2022) for analysis. In total, we identified a total of 8,154 ASVs in fecal samples from 62 individuals. The number of ASVs in AML (ASVs = 5,485) and HC (ASVs = 4,428) was found to be comparable (*p* = 0.070), possibly due to a significant proportion of shared microorganisms (32.07%). However, a notable portion of ASVs was unique to each group, with 67.93% specific to AML patients and 48.66% specific to HC (Figures 1A,B).

We then assessed *α*-diversity, which estimates species richness and evenness of gut microbiota, using the Chao1 and Shannon indices. Neither Chao1 (*p* = 0.074) nor Shannon (*p* = 0.447) showed significant differences between the two groups (Figures 1C,D). Additionally, we evaluated *β*-diversity, which examines species differentiation between different environmental communities, using PCoA and NMDS based on Bray-Curtis metrics. This analysis revealed distinct microbiota profiles between the AML and HC groups (*p* = 0.016, stress = 0.18), indicating a significant shift in microbial composition among newly diagnosed AML patients (Figures 1E–H). We also controlled for potential influences of age and gender on gut microbial diversity in both groups through more detailed comparisons (Supplementary Figures S1A–F).

### Distribution, relative abundance and alterations of gut microbiota at phylum and genus level in newly diagnosed AML patients

**Figure 2:**
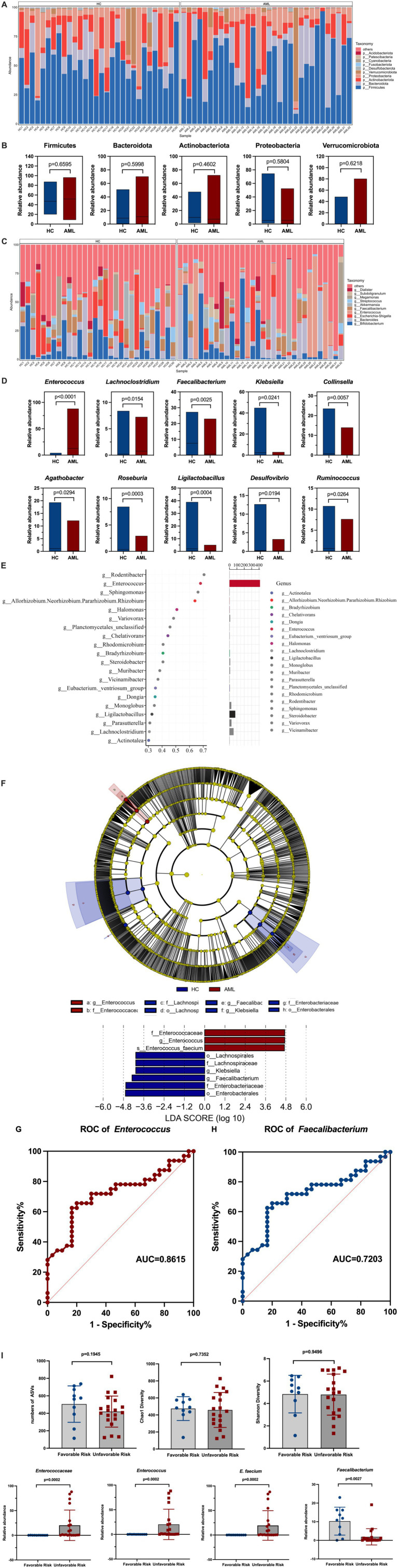
Relative abundance of gut microbiota at phylum and genus level in AML patients. **(A)** Species accumulation histogram of the top 10 most abundant bacteria of all samples at the phylum level. **(B)** Comparison of the relative abundance of the top 5 abundance at the phylum level (Mann–Whitney *U* test). **(C)** Species accumulation histograms of the top 10 most abundant bacterial genera for significantly different samples at the genus level. **(D)** Comparison of the relative abundance for the main detected genera (Mann–Whitney *U* test). **(E)** Random-forest plot. Point plot of species (variables) importance: the horizontal coordinate is a measure of importance, and the vertical coordinate is a species name in order of importance. The bar chart shows the relative abundance of the corresponding species. **(F)** Cladograms and Histograms were generated from LEfSe and LDA scores and shown bacterial taxa that were significantly different in abundance between AML and HC. LDA scores is applied to estimate the effect size. **(G,H)** ROC curve analysis of fecal *Enterococcus* and *Faecalibacterium* in the predictive capacity of AML. **(I)** Changes in the characterization of gut microbiota between the favorable risk (*n* = 10) and unfavorable risk (*n* = 20) group (Mann–Whitney *U* test).

To identify the detailed alterations in bacterial composition, we conducted a comprehensive analysis of relative abundances at both the phylum and genus levels. Initially, we found that the five most prevalent bacterial phylum among AML patients were *Firmicutes* (51.44%), *Bacteroidota* (19.50%), *Actinobacteriota* (15.45%), *Proteobacteria* (8.73%), and *Verrucomicrobiota* (3.94%). In contrast, the top five most abundant bacterial phylum in the HC group were *Firmicutes* (49.5%), *Actinobacteriota* (16.36%), *Proteobacteria* (15.50%), *Bacteroidota* (13.04%), and *Verrucomicrobiota* (4.16%) (Figure 2A). Comparison revealed increased abundance of *Firmicutes* and *Bacteroidota*, while decreased abundance of *Proteobacteria*, *Actinobacteriota* and *Verrucomicrobiota* was observed in AML patients, but there was no statistical difference: *Firmicutes* (*p* = 0.6595), *Actinobacteriota* (*p* = 0.4602), *Proteobacteria* (*p* = 5,804), *Bacteroidota* (p = 5,998), and *Verrucomicrobiota* (*p* = 0.6218) (Figure 2B). Furthermore, at the genus level, a total of 870 bacterial genera were detected across the two groups, of which 197 showed differences in abundance. Among the top 10 abundances of genera with significant differences, *Enterococcus* (*p* < 0.0001) and *Lachnoclostridium* (*p* = 0.0154) were found to be enriched, while *Roseburia* (*p* = 0.0003), *Ligilactobacillus* (*p* = 0.0004), *Faecalibacterium* (*p* = 0.0025), *Collinsella* (*p* = 0.0057), *Desulfovibrio* (*p* = 0.0194), *Klebsiella* (*p* = 0.0241), *Ruminococcus* (*p* = 0.0264), *Agathobacter* (*p* = 0.0294) were reduced in newly diagnosed AML patients, as depicted in Figures 2C,D. Additionally, it was observed that *Enterococcus* shows considerable abundance and importance among the diverse species identified in the Random Forest plot shown in Figure 2E. Importantly, we used LEfSe to excavate differential species serving as gut biomarker in newly diagnosed AML patients. The results confirmed that *Enterococcacea*e (LDA value = 4.726, *p* < 0.00001), *Enterococcus* (LDA value = 4.726, *p* < 0.00001), and *E. faecium* (LDA value = 4.713, *p* < 0.00001) were the most valuable gut biomarker (Figure 2F). ROC curve analyses indicated that *Enterococcus* demonstrated an AUC value of 0.8615, while *Faecalibacterium* yielded an AUC value of 0.7208 (Figures 2H,I). An AUC closer to 1 indicates superior diagnostic efficacy, with values falling within specific ranges denoting different levels of accuracy: excellent (AUC ≥ 0.9), very good (0.8 ≤ AUC < 0.9), good (0.7 ≤ AUC < 0.8), sufficient (0.6 ≤ AUC < 0/7), bad (0.5 ≤ AUC <0.6) and index not useful (AUC <05) (Jahangiri et al., 2019). Consequently, we concluded that *Enterococcus* is a strong predictor of newly diagnosed AML.

We conducted a detailed analysis of the relationship between differential gut microbiota and risk stratification in AML patients. Our results showed no significant differences in ASVs (*p* = 0.1945), Chao1 (*p* = 0.7352), or Shannon (*p* = 0.9496) indices between favorable risk and unfavorable risk patients. However, we observed that the abundance of *Enterococcaceae* (*p* = 0.0002), *Enterococcus* (*p* = 0.0002), and *E. faecium* (p = 0.0002) were significantly higher in un-favorable risk AML patients, while the abundance of *Faecalibacterium* (*p* = 0.0027) was significantly lower in this group (Figure 2I).

### Alterations of fecal microbiota metabolites SCFAs in newly diagnosed AML patients

**Figure 3:**
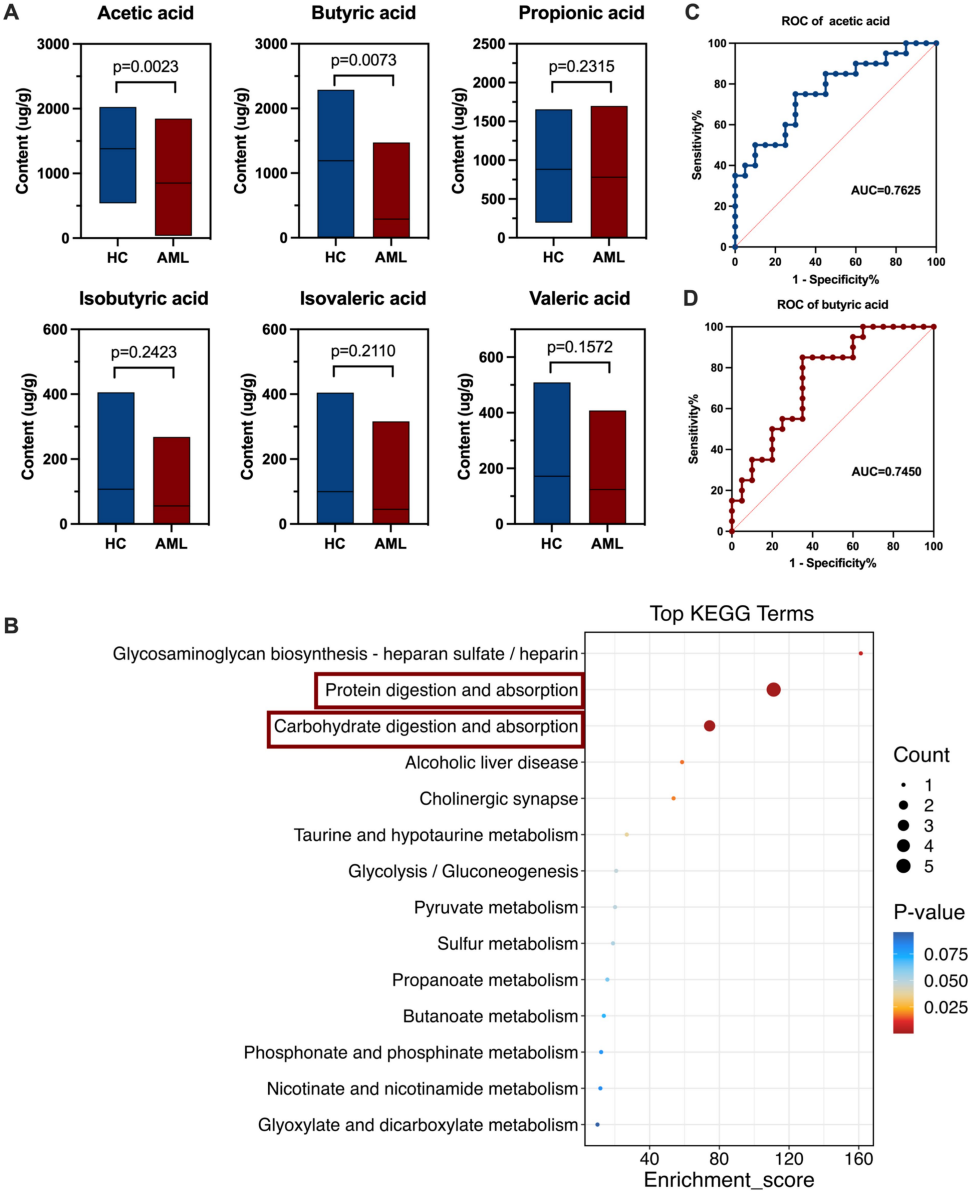
Alterations of fecal microbiota metabolites SCFAs in AML patients. **(A)** The boxplot of SCFAs in fecal samples of AML patients (n = 20) and controls (*n* = 20) (Mann–Whitney U test). **(B)** KEGG Enrichment score (the horizontal coordinate represents enrichment score, and the vertical coordinate represents the names of enriched KEGG metabolic pathways). **(C,D)** ROC curve analysis of fecal butyric acid and acetic acid in the predictive capacity of AML.

Targeted SCFAs metabolomics analysis was employed to quantitatively determine the levels by GC–MS from 40 individuals, comprising 20 newly diagnosed AML patients and 20 healthy controls. Upon comparing the metabolite abundances, we observed significant down-regulation of acetic acid (*p* = 0.0023) and butyric acid (*p* = 0.0073) in AML patients (Figure 3A). Subsequently, we performed further functional analysis based on the Kyoto Encyclopedia of Genes and Genomes (KEGG) metabolite database and found that the differential metabolites butyric acid and acetic acid caused significant differences in the relative abundance of intestinal protein digestion and absorption (*p* < 0.00001, 71.43%, enrichment score = 111.17, DA Score = −1) as well as carbohydrate digestion and absorption (*p* < 0.00001, 42.86%, enrichment score = 74.4, DA Score = −1) (Figure 3B, Supplementary Figures 2A,B). Additionally, ROC curve analysis demonstrated that butyric acid assisted in the predictive of AML with a ROC-plot AUC value of 0.7450, while acetic acid showed a ROC-plot AUC value of 0.7625 (Figures 3B,D). Together, these findings collectively suggest that acetic acid and butyric acid may play pivotal roles in the progression of AML.

### Correlation analysis of gut microbiota and metabolites, hematological parameters in AML patients

**Figure 4:**
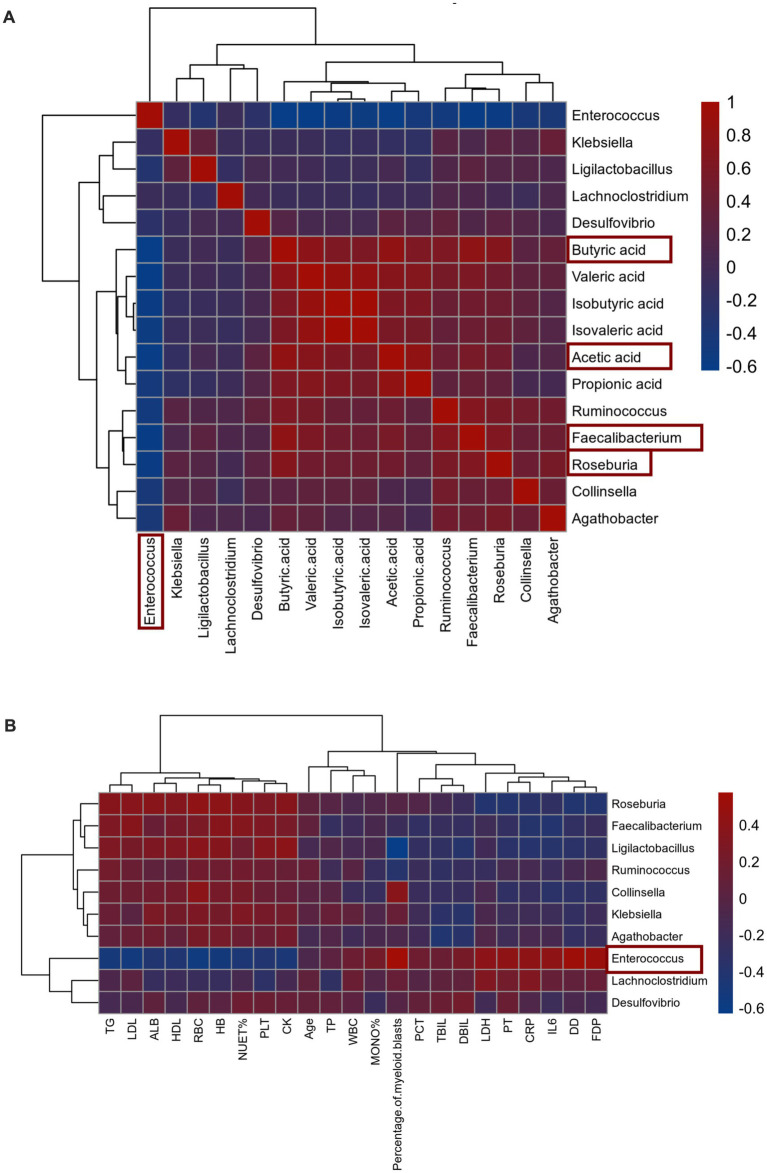
**(A,B)** Heatmap of Spearman correlations between gut microbiota, metabolites and clinical characteristics (red positive correlation, blue negative correlation).

To further elucidate the relationship between gut microbiota, we conducted correlation analysis and confirmed that *Enterococcus* exhibited a negative correlation with beneficial microbiota *Faecalibacterium* (*r* = −0.6036, *p* = 0.000000207) and *Roseburia* (*r* = −0.5628, *p* = 0.00000192) (Figure 4A). Additionally, our metabolite correlation analysis indicated a significant positive correlation between acetic acid and butyric acid (*r* = 0.783, *p* < 0.00001) (Figure 4A). In our investigation of the potential of microbiota in AML, we performed a correlation analysis the correlation analysis between gut microbiota and metabolites, as well as hematological parameters of AML patients, using Spearman correlation analysis. We concentrated on the top 10 most abundant bacteria, significantly different metabolites, and hematological parameters. Ultimately, the results demonstrated that *Enterococcus* was significantly negatively correlated with butyric acid (*r* = −0.626, *p* = 0.00002) and acetic acid (*r* = −0.594, *p* = 0.00007). Conversely, *Faecalibacterium* was significantly positively correlated with acetic acid (*r* = 0.567, *p* = 0.00017) and butyric acid (*r* = 0.792, *p* = 0.00005) (Figure 4A).

Furthermore, among various clinical characteristics, *Enterococcus* showed a positive correlation with DD (*r* = 0.540, *p* < 0.001), FDP (*r* = 0.508, *p* < 0.001), DBIL (*r* = 0.251, *p* = 0.049), LDH (*r* = 0.403, *p* = 0.003), IL-6 (*r* = 0.424, *p* = 0.004), CRP (*r* = 0.417, *p* = 0.005), PT (*r* = 0.425, *p* < 0.001), and the percentage of myeloid blasts (*r* = 0.580, *p* < 0.001). Conversely, it demonstrated a negatively correlated with PLT (*r* = −0.412, *p* < 0.001), RBC (*r* = −0.556, *p* < 0.001), HB (*r* = −0.521, *p* < 0.001), ALB (*r* = −0.421, *p* < 0.001), TG (*r* = −0.507, *p* < 0.001), LDL (*r* = −0.529, *p* < 0.001), HDL (*r* = −0.438, *p* < 0.001), CK (*r* = −0.472, *p* < 0.001), and NEUT% (*r* = −0.457, *p* < 0.001) (Figure 4B). These findings further suggested *Enterococcus* may play an significant role in the progression of AML.

### Isolation and identification of intestinal colonization *E. faecium* in newly diagnosed AML patients

**Figure 5:**
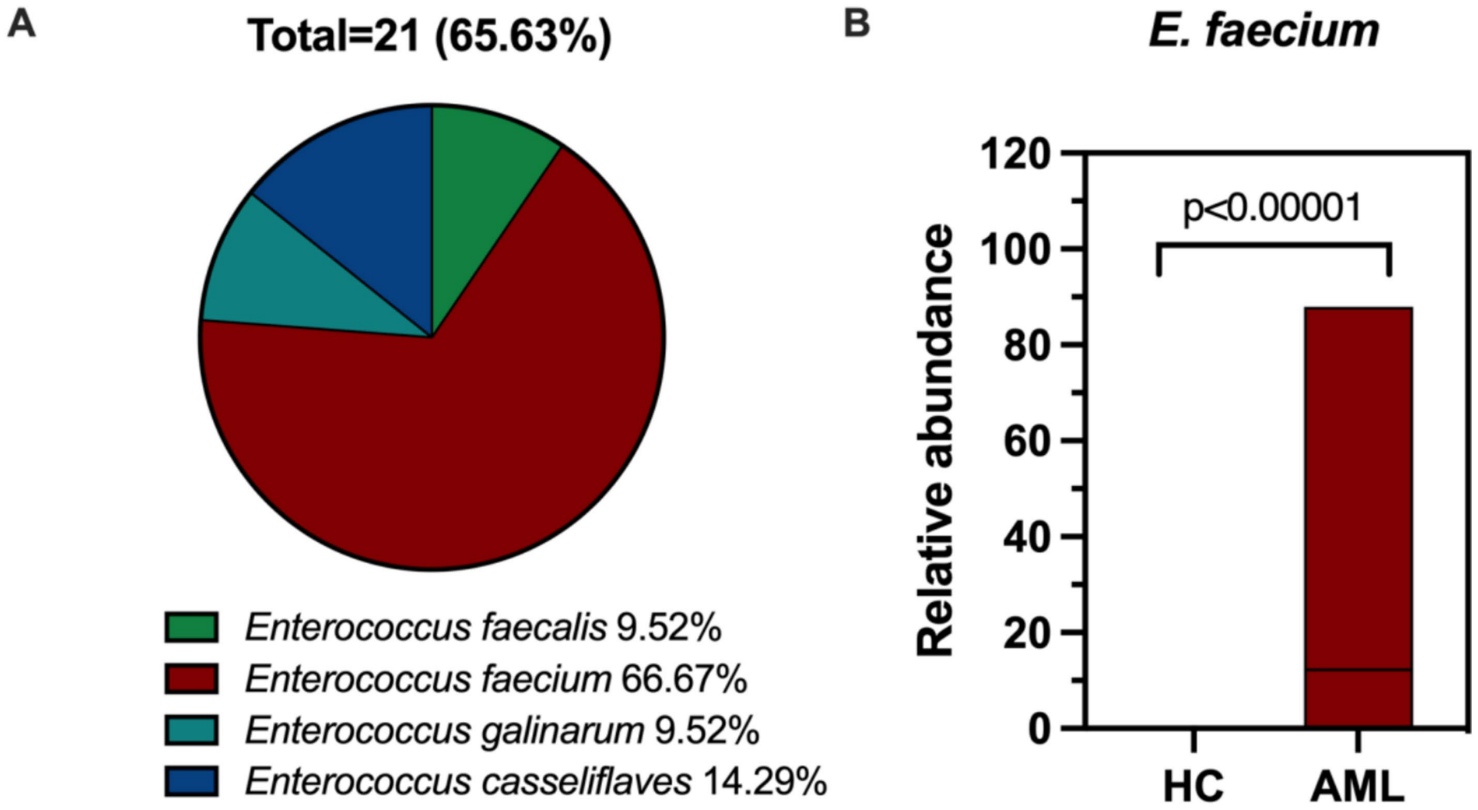
**(A)** Composition ratio of intestinal colonizing *Enterococcus*. **(B)** The boxplot of *E. faecium* in fecal samples of AML patients (*n* = 32) and controls (*n* = 30).

Based on the findings of gut microbiological analysis, we inoculated all fecal samples onto Columbia Blood Agar plates for isolation and culture. The results indicated that no isolate of *Enterococcus* was isolated from any of the 30 healthy individuals, meaning that no intestinal colonization was detected in this group. In contrast, 21 isolates were isolated from 21 out of 32 AML patients, reflecting a colonization rate of 65.63%. This included 2 isolates of *E. faecalis* (9.52%), 2 isolates of *Enterococcus galinarum* (*E. galinarum*, 9.52%), 3 isolates of *Enterococcus casseliflaves* (*E. casseliflaves*, 14.29%), and 14 isolates of *E. faecium* (66.67%) (Figure 5A). Further analysis at the species level using 16S rRNA sequencing revealed that only *E. faecium* showed considerable differences between the AML and HC groups (*p* < 0.00001) (Figure 5B). In contrast, the following species did not show significant differences: *E. faecalis* (*p* = 0.1585), *Enterococcus asini* (*p* = 0.1409), Enterococcus avermectinum (*p* = 0.9671), *Enterococcus canis* (*p* = 0.1674), *E. casseliflaves* (*p* = 0.069), *Enterococcus diestrammenae* (*p* = 0.3017), *E. galinarum* (0.4953), *Enterococcus hirae* (p = 0.1674), *Enterococcus raffinosus* (p = 0.1674), *Enterococcus rivorum* (*p* = 0.3329) and *Enterococcus saccharolyticus* (*p* = 0.3017) (Supplementary Table S1). From this, it is evident that the profile of the isolates we isolated aligns well with the outcomes of the gut microbiome sequencing.

### Antimicrobial susceptibility test, AMR determinants, and STs of *E. faecium*

A total of 14 colonized isolates of *E. faecium* were tested for antimicrobial susceptibility, and chosen for NGS. The antimicrobial susceptibility profiles demonstrated that the overwhelming majority of isolates (92.85%, 13/14) displayed multidrug resistance phenotypes, as indicated in Table 3.

**Table 3:** Phenotypic profile of antimicrobial resistance in *E. faecium* isolates (*n* = 14).

Antimicrobial agent	Sensitivity (*n*, %)	Intermediary (*n*, %)	Resistance (*n*, %)
Gentamicin High Level (GEN-HL)	7 (50)	0 (0)	7 (50)
Streptomycin High Level (STR-HL)	10 (71.43)	0 (0)	4 (28.57)
Ciprofloxacin (CIP)	1 (7.15)	0 (0)	13 (92.85)
Levofloxacin (LVX)	1 (7.15)	0 (0)	13 (92.85)
Erythromycin (ERY)	1 (7.15)	1 (7.15)	12 (85.7)
Quinupristin/dalfopristin (QD)	13 (92.85)	1 (7.15)	0 (0)
Linezolid (LZD)	14 (100)	0 (0)	0 (0)
Vancomycin (VAN)	14 (100)	0 (0)	0 (0)
Tetracycline (TET)	5 (35.71)	0 (0)	9 (64.29)
Tigecycline (TIG)	14 (100)	0 (0)	0 (0)

Regarding aminoglycosides, high-level gentamicin resistance (HLGR) phenotype was observed in 50% of *E. faecium* isolates (7/14), while high-level streptomycin resistance (HLSR) was identified in 28.57% of *E. faecium* isolates (4/14). Additionally, three *E. faecium* isolates demonstrated both HLGR and HLSR phenotypes. This resistance is primarily attributed to the high detection rates of the AAC (6′)-Ii gene (100%) and AAC (6′)-Ie-APH (2″)-Ia (92.86%). Furthermore, 13 isolates (92.85%) showed resistance to ciprofloxacin (CIP) and levofloxacin (LVX), while 12 isolates (85.7%) exhibited a significant resistance phenotype to erythromycin (ERY), which is associated with the presence of the drug-resistance genes efrA (100%) and efmA (100%). Tetracycline (TET) resistance was identified in the majority of *E. faecium* isolates (64.29%), with the major resistance gene being tetM (64.29%). However, all isolates remained susceptible to tigecycline (TIG), linezolid (LZD), and vancomycin (VAN), with a notable susceptibility (92.85%) to quinupristin/dalfopristin (Q/D) (Table 4).

**Table 4:** The presence of virulence-associated genes in the study 14 *E. faecium* isolates.

Class	VFs	Related genes	AML Patients colonized with *E. faecium*													
			1	2	4	5	7	13	14	15	19	26	29	30	31	32
Adherence	Acm	acm	+	+	+	+	+	+	+	+	+	+	+	+	+	+
		M7W_2,305														+
	Ebp pili	ebpC												+		
	EfaA	EFAU085_00431	+	+	+	+	+	+	+	+	+	+	+	+	+	+
	Esp	esp	+							+	+	+	+	+	+	
	Fbp	sagA	+	+	+	+	+	+	+	+	+	+	+	+	+	+
		fss3	+							+	+	+	+	+	+	
	Scm	scm				+					+					
	SgrA	sgrA	+	+		+	+	+	+	+	+	+	+	+		
Antiphagocytosis	Capsule	EFAU085_01747	+	+	+	+	+	+	+	+	+	+	+	+	+	+
Biofilm	BopD	EFAU004_00405	+		+			+		+	+	+	+	+	+	
		EFAU085_00344		+		+	+		+							+

**Figure 6:**
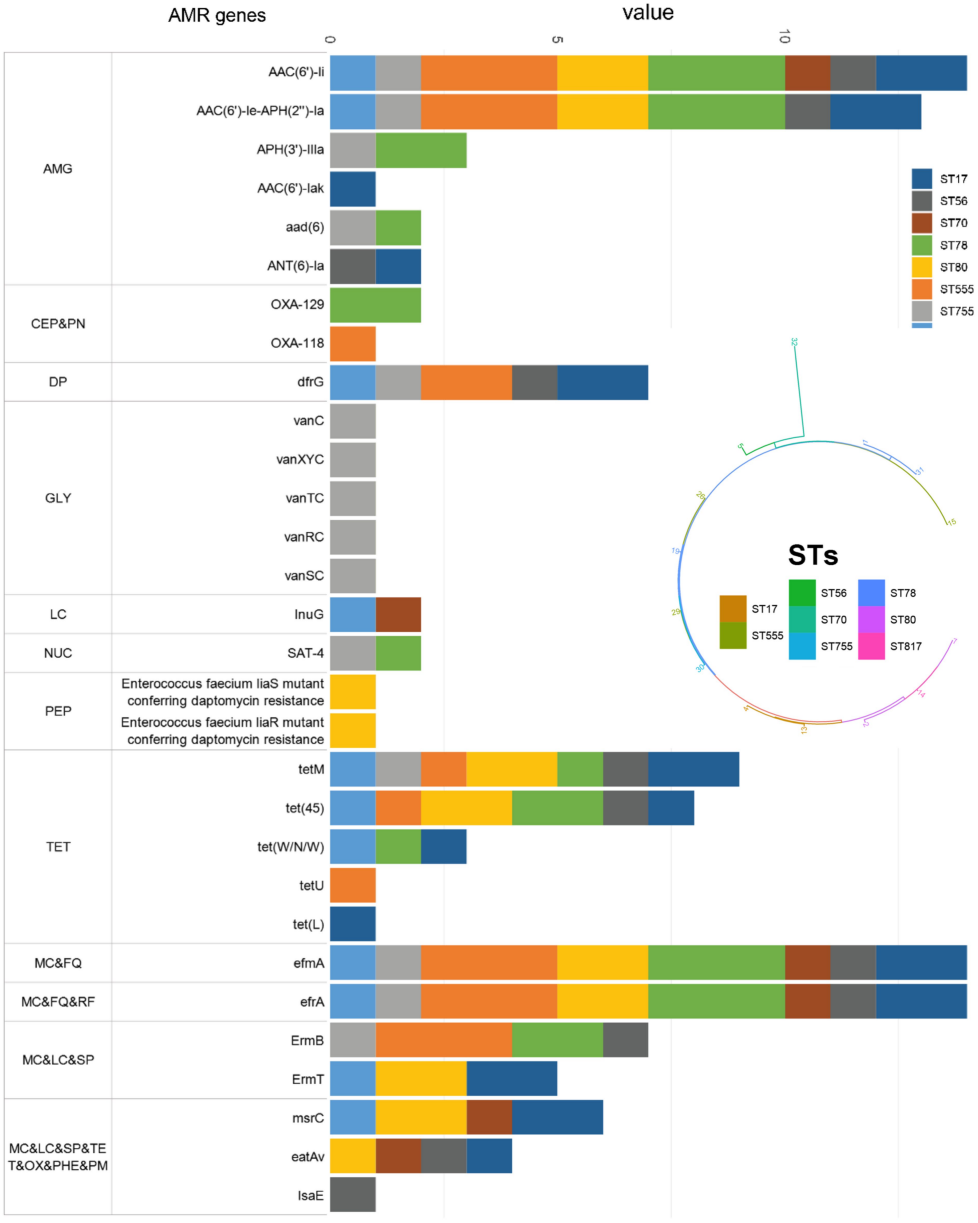
Distribution of AMR genes in the study 14 *E. faecium* clinical isolates according to STs. AMG: aminoglycoside antibiotic; CEP: cephalosporin; PN: penam; DP: diaminopyrimidine antibiotic; GLY: glycopeptide antibiotic; LC: lincosamide antibiotic; NUC: nucleoside antibiotic; PEP: peptide antibiotic; TET: tetracycline antibiotic; MC; macrolide antibiotic; FQ; fluoroquinolone antibiotic; RF: rifamycin antibiotic; SP: streptogramin antibiotic; OX: oxazolidinone antibiotic; PHE: phenicol antibiotic; PM: pleuromutilin antibiotic.

Different colours represent different STs. Since the information on STs of different isolates is represented in Figure 6.

Among the 30 antibiotic-resistant genes identified in *E. faecium* isolates and their highest detection rates were, in order, AAC (6′)-Ii (100%), efmA (100%), efrA (100%), AAC (6′)-Ie-APH (2″)-Ia (92.86%) and tetM (64.29%). The STs of *E. faecium* isolates were determined using the *E. faecium* scheme in PubMLST (El Zowalaty et al., 2023). A total of 8 distinct STs were identified, with ST555 (*n* = 3, 21.43%) and ST78 (*n* = 3, 21.43%) being the highest frequency, followed by ST80 (*n* = 2; 14.28%) and ST817 (*n* = 2; 14.28%) (Figure 6).

### VFs and virulence-associated genes of *E. faecium*

The investigation into the VFs of *E. faecium* isolates revealed the presence of several virulence genes associated with adherence, antiphagocytosis, and biofilm formation (Table 4).

The VFs related to adhesion include Acm (a collagen adhesin), Ebp pili (endocarditis and biofilm-associated pili) (Cebeci, 2024), EfaA (*E. faecalis* antigen A), Esp (enterococcal surface protein), Fbp (fibronectin-binding proteins), Scm [collagen-binding microbial surface components recognizing adhesive matrix molecule (MSCRAMM), SgrA (a cell wall anchored protein)]. We identified two genes encoding Acm: acm, which had a carrying rate of 100%, and M7W-2305, which had a carrying rate of 7.14%. Only one isolate contained the ebpC gene encoding Ebp pili. All isolates carried the EFAU085_00431 gene encoding EfaA, and 50% (7/14) of the isolates contained the esp. gene encoding Esp. Additionally, we detected two genes, sagA, and fss3, that encode Fbp, with carrying rates of 100 and 50%, respectively. 14.29% (2/14) isolates carried the scm gene encoding Scm and 78.57% (11/14) isolates carried the sgrA gene encoding SgrA. Among the VFs associated with antiphagocytosis, the most significant is the capsule encoded by the EFAU085_01747 genes, which had a 100% carriage rate. Another important VF involved in biofilm formation is BopD (biofilm on plastic surfaces, a putative sugar-binding transcriptional regulator) for biofilm formation. The ability of *E. faecium* to form biofilms is a notable characteristic of its pathogenicity (Wei et al., 2023). In this study, 64.29% of the isolates carried the EFAU004_00405 gene and 35.71% carried the EFAU085_00344 gene encoding BopD. The pathogenesis of *E. faecium*, attributed to these VFs, contributes to its adherence, colonization, and invasion of the host while causing damage.

## Discussion

Dysregulation of the gut microbiota has been implicated in the pathogenesis of various diseases, such as metabolic disorders, inflammatory disease, and cancer (Yu et al., 2021). As the most significant hematologic tumor, the relationship between AML and gut microbiota is gaining increasing attention. IC, combined with broad-spectrum antibiotics in AML treatment, leads to gut microbiota dysbiosis and temporarily impairs gut barrier function, which can promote pathological conditions and increase the likelihood of complications (Malard et al., 2021). For instance, a severe reduction in the levels of *Odoribacter splanchnicus* and *Gemminger formicilis* in AML patients has been associated with impaired gut barrier function and loss of body weight (Pötgens et al., 2023). Additionally, the levels of *E. eligens*, which was reduced threefold in AML patients, showed a strong correlation with muscle strength (Pötgens et al., 2024). However, most studies conducted so far have primarily focused on the treatment phase of AML. In this study, the objective was to explore the alterations of gut microbiota of patients with a primary diagnosis of AML.

We conducted a preliminary exploration of significant changes in gut microbiota among newly diagnosed AML patients compared to individuals without the condition. While the overall gut microbiota (*α*-diversity) was not significantly altered, the structure and predominant types of microbiota (*β*-diversity) exhibited significant changes. Therefore, the reduction in α-diversity is not universal among newly diagnosed AML patients, and we hypothesize that it may occur more frequently during treatment, such as chemotherapy, immunotherapy, and antibiotic tuse, which aligns with findings from other studies (Xu et al., 2023; Reyman et al., 2022). In our phylum-level abundance analysis, we observed that the abundance of the *Proteobacteria* was nearly two times higher in the AML group compared to the HC group. Our study identified the top 30 differentially abundant genera, which included seven genera of *Proteobacteria*. Among these, five genera were down-regulated: *Klebsiella* (*p* = 0.0266), *Desulfovibrio* (*p* = 0.0193), *Citrobacter* (*p* = 0.0362), *Ralstonia* (*p* = 0.044), and *Enterobacter* (*p* = 0.0024). The relative abundance of *Desulfovibrio* was positively correlated with beneficial bacterial genera, including *Ruminococcus*, *Akkermansia*, *Roseburia*, *Faecalibacterium* and *Bacteroides*, which suggests that *Desulfovibrio* is associated with healthy hosts in some populations (Chen et al., 2021). In contrast, two genera, *Brevundimonas* (*p* = 0.019) and *Steroidobacter* (*p* < 0.0001), were up-regulated (Supplementary Figure S3). However, when analyzing the overall phylum level, no statistical differences were observed. Analyzing at the genus level, we confirmed that *Enterococcus* and *Lactobacillus* were enriched in newly diagnosed AML patients, particularly *Enterococcus*. In contrast, beneficial bacteria such as *Faecalibacterium*, *Collinsella*, *Liilactobacillus,* and *Roseburia* were found to be significantly decreased. Based on a macrogenomic analysis of more than 7,900 human samples, a recent study suggests that the ideal mean and median relative abundance of *Faecalibacterium* in healthy adults may be 6.5 and 4.8%, respectively (Martín et al., 2023). In the present study, the mean and median values of *Faecalibacterium* in the healthy population were 8.49 and 7.54%, which were slightly higher than the ideal abundance as it was correlated with age, lifestyle, geographic location, and disease, among other things (De Filippis et al., 2020), whereas the mean and median values in patients with AML were much lower than the ideal abundance, at 4.39 and 0.74%, respectively. A randomized trial conducted by Rashidi et al. also reported an increase in *Enterococcus* and a decrease in *Roseburia*, *Faecalibacterium*, and *Collinsella* in AML patients at baseline. However, after undergoing a fecal microbiota transplantation (FMT), patients saw a reduction in *Enterococcus* and a return of *Collinsella*, along with an increase in overall microbiota diversity (Rashidi et al., 2023). These findings support the idea that targeting the dominant microbiota may be a viable strategy for managing AML progression. Butyric acid producing bacteria (*Rumminococcus*, *Roseburia*, *Clostridium* and *Eubecterium Limosum*), *Bifidobacterium* that cross-fed butyric acid, *Akkermensia* and *Enterococcus* have been reported to play a significant role in patients with hematological malignancies, with *Enterococcus*, defined as a “pro-inflammatory bacterium,” was negatively associated with butyric acid-producing *Roseburia*, which is also consistent with our findings (Yu et al., 2021; Malard et al., 2021). Furthermore, we have demonstrated that *Enterococcus* and *Faecalibacterium* are important intestinal biomarkers for screening patients with AML. Even more valuable is the fact that changes in the characteristic microbiota also show significant value in risk stratification.

A large number of reports indicate that SCFAs, originated from the metabolism of gut microbiota (Wu et al., 2023; Guo et al., 2020), more than 95% of the SCFAs in the human colon lumen are composed of acetatic acid, propionate, and butyric acid. It is well known that butyric acid is mainly derived from Faecalibacterium and Roseburia (Machiels et al., 2014) and acetatic acid is mainly derived from Akkermansia, Bacteroides, Bifidobacterium, Prevotella, Ruminococcus, and Streptococcus (Wang et al., 2023). Our results revealed that the abundance of these bacteria was significantly reduced in AML patients. SCFAs, mainly butyric acid, promote epithelial barrier integrity and permeability by upregulating proteins encoding tight junction proteins (e.g., claudin-1, zonula occludens-1, and occludin), strengthening the mucus layer of the intestinal epithelium by increasing mucin 2 expression, and modulating intestinal oxidative stress (Wang et al., 2022; Fusco et al., 2023). SCFAs exert anti-inflammatory functions by modulating immune cell chemotaxis, reactive oxygen species (ROS), and cytokine release (decrease IL-6, IL-8 and increase IL-10, TNF-*α*) (Inamoto et al., 2023), reduce DNA damage during radiation (a recognized cause of leukemia) injury and butyric acid serves as a colonic fuel sources, fosters immunoregulation (Guo et al., 2020; Liu et al., 2024). SCFAs can promote the elongation of dendritic cell line (DC2.4 cells) and mouse bone marrow-derived dendritic cells by inhibiting HDAC, stimulating the SFK/PI3K/Rho family pathways, and activating actin polymerization, thereby enhancing the uptake and expression of antigens by dendritic cells (DCs) (Inamoto et al., 2023). Research indicates that immunogenic dendritic cells from donors with higher α-diversity of gut microbiota and higher abundance of SCFAs and SCFA-producing bacteria exhibited lower expression of CD1a, CD86, CD40, pro-inflammatory cytokines, and immunogenicity. These results highlight the importance of the gut microbiota in promoting the differentiation of donor precursor cells to immunogenic DCs capable of effectively engaging in cancer therapy. This discovery may provide new ideas for future DC-based cancer therapy suggesting that increased microbial diversity and SCFA abundance could be critical factors in the development of novel immunotherapies (Radojević et al., 2021). Through correlation analysis, we discovered a significant negative correlation between *Enterococcus* and the percentage of myeloid blasts, as well as butyric acid and acetatic acid levels in patients. In contrast, *Faecalibacterium* exhibited the exact opposite. Therefore, we hypothesized that the increased colonization of *Enterococcus* in the intestines of AML patients leads to microbiota dysbiosis, resulting in a decrease in the number of beneficial bacteria such as *Faecalibacterium* and *Roseburia,* and indirectly causing a decrease of SCFAs (mainly butyric acid and acetatic acid). Further, we analyzed the KEGG database and found significant differences in the processes involved in protein digestion and absorption, carbohydrate digestion and absorption, and fatty acid synthesis. Therefore, we hypothesize that *Enterococcus* influences AML progression by indirectly causing a decrease in acetatic acid and butyric acid synthesis.

To further observe the pathogenic characteristics of *E. faecium* at the species level, we isolated 21 isolates of *E. faecium* from the feces of 32 AML patients, whereas no isolate was isolated from 30 individuals, where the dominant isolate was *E. faecium* (66.67%). In recent years, with the use of broad-spectrum antibiotics, immunosuppressants, and the increase of invasive manipulation, *E. faecium* infections have gradually become the predominant pathogen of *Enterococcus* (De Oliveira et al., 2020). The results of the present study further indicate that *E. faecium* became the primary species colonizing the intestines of AML patients. As we all know, *E. faecalis* and *E. faecium* are the two most predominant *Enterococcus* in the gastrointestinal tract and an important group of opportunistic pathogens in humans (Aung et al., 2023; Moon et al., 2023), which frequently cause periodontal, wound, urinary tract infections (Codelia-Anjum et al., 2023), intravascular catheter infections (Muff et al., 2021), bacteremia (Rosselli Del Turco et al., 2021), and nosocomial infections, accounting for 14.7% of all healthcare-associated infections in adults (Șchiopu et al., 2023). They are most relevant to human disease and carry many inherent and acquired AMR and virulence genes (Rogers et al., 2021). In Mancuso et al., 2021, the WHO released a list of pathogens for which the development of new antibiotics is urgently required, in order to focus and guide research and development efforts. Among this extensive list, the ESKAPE pathogens—comprising *E. faecium, Staphylococcus aureus, Klebsiella pneumoniae, Acinetobacter baumannii, Pseudomonas aeruginosa* and *Enterobacter species*—were assigned “priority status” (De Oliveira et al., 2020). In addition, especially *E. faecium*, which can acquire antibiotic resistance through chromosomal mutations or gene exchange, there has been a clear shift toward pathogens with multi-drug resistance (Hammerum et al., 2024; Krause et al., 2022; Freitas et al., 2021). This study revealed the high resistance rates of *E. faecium* isolates to AMG, MC, and quinolone antibiotics. TIG, LZD, and VAN are critically important for the treatment of *Enterococcus*. Fortunately, no resistant phenotype was detected. QD is a streptogramin combination and an important treatment option for VAN-resistant *E. faecium* infections in humans. In the present study, *E. faecium* was found to be highly susceptible (92.85%) to QD.

The pathogenesis of *Enterococcus* is attributed to a diverse range of VFs. The identification of VFs is crucial in evaluating bacterial pathogenicity, as it contributes to the attachment, colonization, and invasion of host tissues, and also affects the host’s immune response and production of enzymes and toxins, which enables microorganisms to invade and harm the host (Cebeci, 2024; Kiruthiga et al., 2020). In *E. faecium*, Acm is the most well-characterized MSCRAMM, presumably enhancing the ability to survive and/or cause infection in the clinical setting (Nallapareddy et al., 2006; García-Solache and Rice, 2019). The acm gene is predominantly present in 100% of the analyzed isolates in this study. Ebp pili are well-defined cell wall-attached surfaces and can facilitate adhesion to abiotic and biotic surfaces (Choo et al., 2023; Ogrodzki et al., 2017). Only one isolate carried the ebpC gene encoding Ebp pili. EfaA is the most important adhesion protein in *Enterococcus* and plays a vital role in adhesion to eukaryotic cells and surfaces along with the colonization of host tissues (Ghazvinian et al., 2024), and all isolates carried the EFAU085_00431 gene encoding EfaA. Esp., Scm, and SgrA, as the surface-anchored proteins of *E. faecium,* are also important virulence determinants (García-Solache and Rice, 2019; Gao et al., 2018; Taglialegna et al., 2020; Rotta et al., 2022; Kim and Kim, 2022). In the present study, we found that 50% (7/14) of the isolates carried esp. gene encoding Esp., only 14.29% (2/14) isolates carried scm gene encoding Scm and 78.57% (11/14) isolates carried sgrA gene encoding SgrA. Furthermore, EFAU085_01747, a capsule-producing gene associated with anti-phagocytosis, was harbored in all isolates of the current study. Many pathogenic bacteria preserve capsular polysaccharide encoding genes to evade phagocytosis and contribute a significant role in pathogenesis through immune evasion (Akter et al., 2023). Meanwhile, we discovered that 64.29% of isolates carried the EFAU004_00405 gene and 35.71% of isolates carried the EFAU085_00344 gene encoding BopD, which is found to be necessary for biofilm formation (Creti et al., 2006; Espíndola et al., 2021). The ability to attach to host cells and form biofilms makes them more resistant to antibiotic killing and phagocytic attack, which is related to their pathogenic potential and ability to cause disease (García-Solache and Rice, 2019). The high frequency of detection of these VFs, which may contribute to its success as a pathogen, underwrite the pathogenic potential and pathogenicity of *E. faecium*, indirectly demonstrating that increased intestinal colonization of *E. faecium* is closely linked to AML disease progression.

## Conclusion

In conclusion, the structure and abundance of gut microbiota in patients with newly diagnosed AML were significantly altered. In particular, there was a significant increase in the opportunistic pathogen *Enterococcus* and a marked decrease in the beneficial bacteria *Roseburia, Ligilactobacillus, Faecalibacterium*. Interestingly, the metabolites butyric acid and acetatic acid were significantly downregulated. We hypothesize that increased colonization of *Enterococcus* leads to gut microbiota dysbiosis, which indirectly results in a decrease in specific butyric acid and acetatic acid-producing bacteria, leading to a significant negative correlation between *Enterococcus* and butyric acid and acetatic acid. These indicators are valuable intestinal predictive biomarkers for AML patients. Pathogenetic studies of isolated intestinal colonized *E. faecium* confirmed that the colonization rate of *E. faecium* was significantly increased and it carried multi-drug resistance and high virulence genes. Therefore, we hypothesize that *Enterococcus* plays an important role in the disease progression of AML, which is the direction and focus of our subsequent studies.

The study also presents limitations. We used strict inclusion and exclusion criteria to ensure cohort consistency, but this inherently limits the study population and may restrict the generalizability of the results. In addition, due to the complexity of individualized treatment regimens for patients, our research is currently limited to horizontal clinical studies; longitudinal clinical studies require larger clinical samples. Finally, our study can only demonstrate that gut microbiota dysbiosis, especially colonization by *E. faecium*, has increased in untreated AML patients, so we can only hypothesize that *E. faecium* plays an important role in AML disease progression. Therefore, a mechanism study will be our subsequent research direction and focus.

## Data Availability

The datasets generated and analyzed during the current study are available under accession number PRJNA1159986 (https://www.ncbi.nlm.nih.gov/bioproject/PRJNA1159986).

## Footnotes

^1^ https://github.com/rrwick/Unicycler

^2^ https://card.mcmaster.ca/

^3^ https://pubmlst.org/

^4^ https://www.mgc.ac.cn/cgi-bin/VFs/genus.cgi?Genus=*Enterococcus*
