# Supplementary material for: Significant changes in gut microbiota and SCFAs among patients with newly diagnosed acute myeloid leukemia
Source: Front Microbiol. 2025 Apr 1;16:1559033. doi: 10.3389/fmicb.2025.1559033 (PMC11997447; doi:10.3389/fmicb.2025.1559033)
Supplement: Supplementary file 1 [file Data_Sheet_1.docx]

**Supplementary materials**


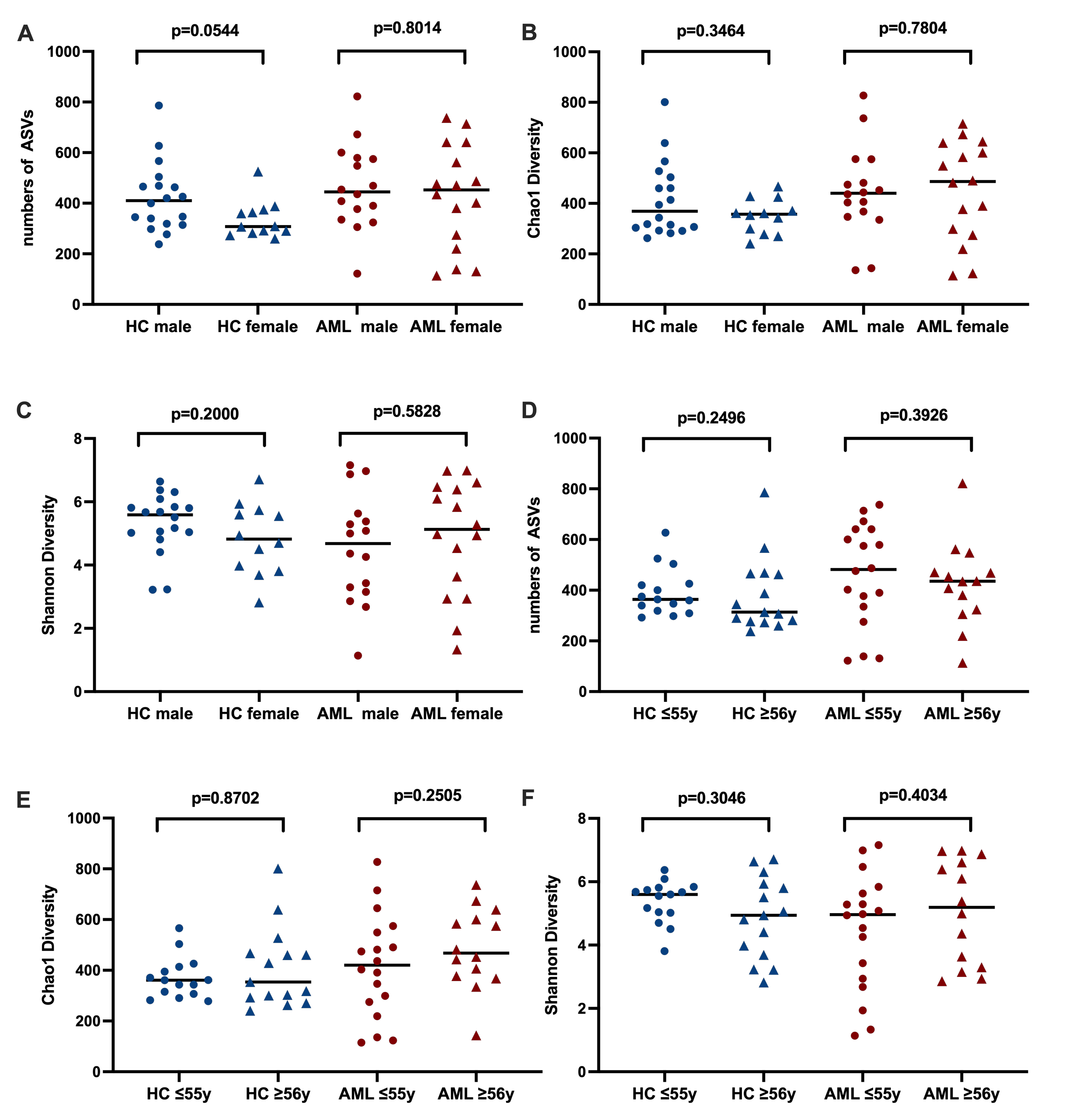


Supplementary Fig. 1A-F The ASVs sequences, Chao1 and Shannon index of gut microbiota diversity in newly diagnosed AML patients and HC group based on different age and sex stratification.


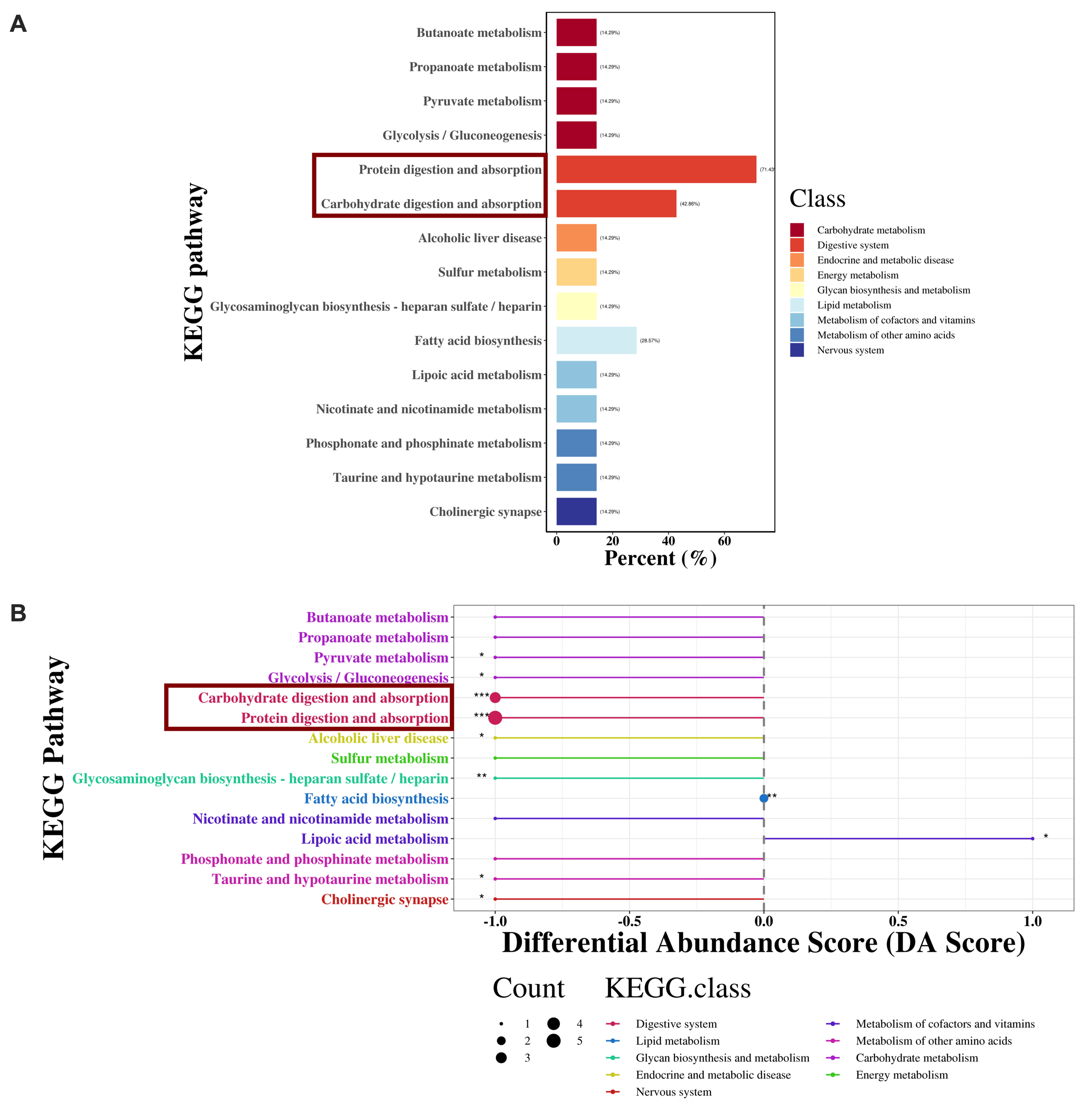


Supplementary Fig. 2A-B (A) KEGG Classification (the horizontal coordinate represents the percentage of the number of annotated differential metabolites in one pathway to all annotated differential metabolites, and the vertical coordinate represents the names of enriched KEGG metabolic pathways). (B) Differential Abundance Score (the horizontal coordinate represents the DA Score (1 indicates up-regulated, -1 indicates down-regulated) and the vertical coordinate represents the KEGG metabolic pathway name). Larger dots indicate a higher number of differential metabolites in the pathway.


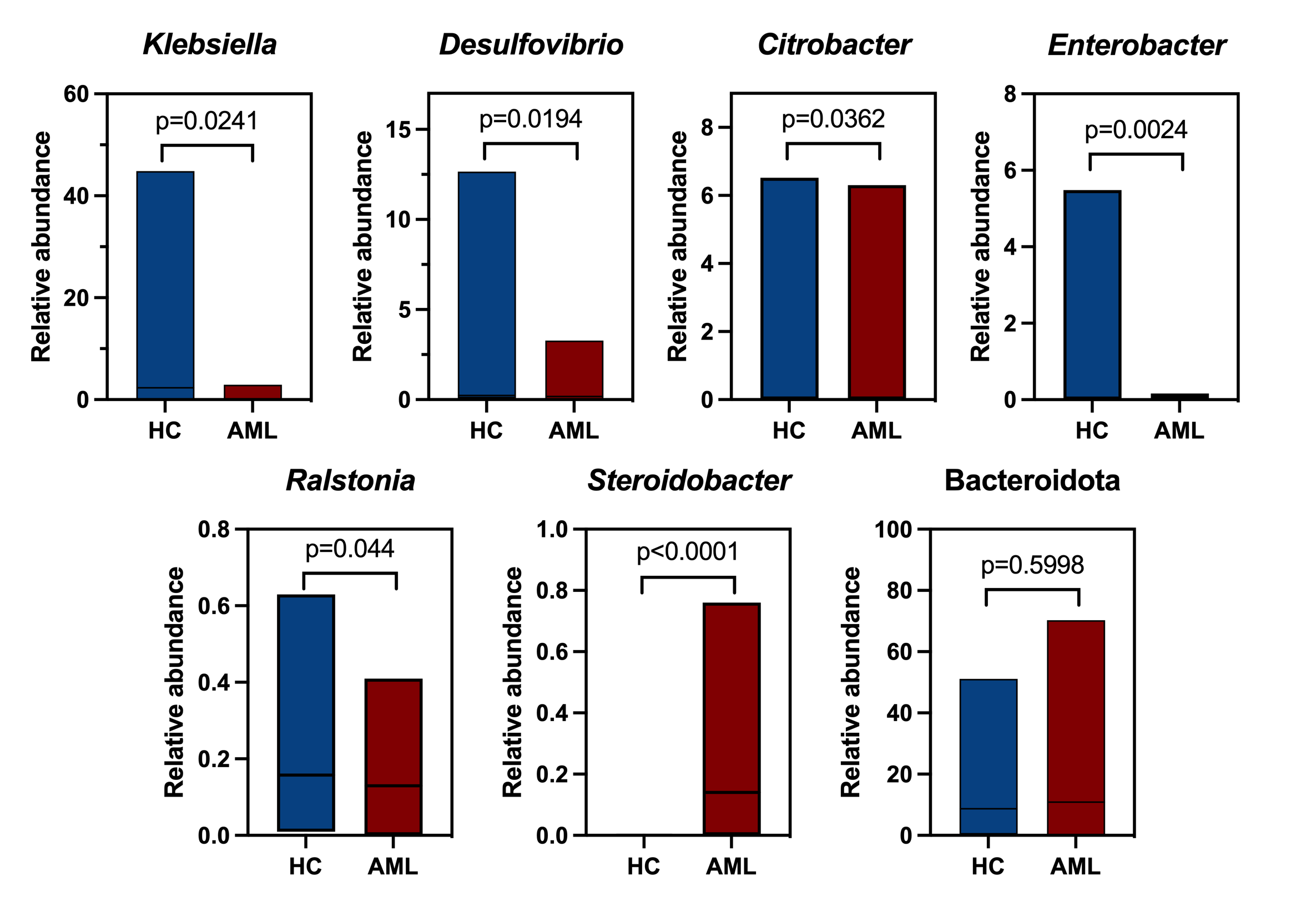


Supplementary Fig. 3 The top 30 genera in abundance included 7 of the Proteobacteria, with 5 genera downregulated in abundance and 2 genera upregulated in AML patients.

Supplementary Table 1 Comparative results of different *Enterococcus* abundances between AML and HC groups.

| Species | p value | significance | regulation |
| --- | --- | --- | --- |
| s__Enterococcus_asini | 0.14 | no | down |
| s__Enterococcus_avium | 0.97 | no | up |
| s__Enterococcus_canis | 0.17 | no | up |
| s__Enterococcus_cecorum | 0.07 | no | down |
| s__Enterococcus_diestrammenae | 0.30 | no | down |
| s__Enterococcus_faecalis | 0.16 | no | up |
| s__Enterococcus_faecium | <0.0001 | yes | up |
| s__Enterococcus_gallinarum | 0.50 | no | down |
| s__Enterococcus_hirae | 0.17 | no | up |
| s__Enterococcus_raffinosus | 0.17 | no | up |
| s__Enterococcus_rivorum | 0.33 | no | up |
| s__Enterococcus_saccharolyticus | 0.30 | no | down |
